# The Effectiveness of Superficial Drain to Reduce Surgical Site Infection in Colorectal Surgery

**DOI:** 10.7759/cureus.17232

**Published:** 2021-08-16

**Authors:** Turki A Alsafrani, Abdullah A Alabbasi, Albara A Dabroom, Moayad M Alhothali, Khalid A Alresini, Ghaleb A Aboalsamh, Ahmed K Abdelhady

**Affiliations:** 1 Orthopedics, King Saud Bin Abdulaziz University for Health Sciences, College of Medicine-Western Region, Ministry of National Guard Health Affairs, Jeddah, SAU; 2 Medicine, King Saud Bin Abdulaziz University for Health Sciences, Jeddah, SAU; 3 Orthopaedics, King Saud Bin Abdulaziz University for Health Sciences, Jeddah, SAU; 4 Department of Medicine, King Saud Bin Abdulaziz University for Health Sciences College of Medicine, Jeddah, SAU; 5 Department of Surgery, King Abdulaziz Medical City, Jeddah, SAU

**Keywords:** surgical site infection, subcutaneous drain, colorectal surgery, superficial ssi, surgical drains

## Abstract

Background

The incidence of surgical site infection (SSI) in colorectal surgery is high, which can complicate and delay postoperative recovery. This study mainly aims to evaluate the efficacy of subcutaneous drains in decreasing superficial surgical site infection in colorectal surgery patients.

Study design

This is a retrospective cohort study that included patients over 16 years old who underwent colorectal surgery from the 1st of January 2015 till the 31st of December 2020. Patients were divided into two groups, with and without a subcutaneous drain. The incidence of superficial SSI was measured as the primary objective, and the incidence of other complications like seromas, hematomas, and wound dehiscence was measured as the secondary objectives or outcomes. Chi-square and Fisher’s exact were used to analyze the data, and a p-value less than 0.05 was accepted for significance.

Results

A total of 208 patients who underwent colorectal surgery in our hospital were included. Of these, 29 had a subcutaneous drain, and 179 did not have a subcutaneous drain. Although the incidence of dehiscence was higher in the drain group, the overall incidence of superficial SSI (20.7%) and seroma/hematoma (3.4%) in patients with subcutaneous drains was lower than without subcutaneous drains (25.7% and 7.8%, respectively). However, no statistical significance was found between drain presence and complications.

Conclusion

In conclusion, this study demonstrated a lower incidence of superficial SSI and seroma/hematoma in patients with a subcutaneous drain than those who did not have a drain.

## Introduction

Surgical site infection (SSI) is a common complication following surgeries, and it forms a substantial part of nosocomial infections with a percentage of 38% [1,2]. SSI develops in around 2-5% of patients undergoing surgery, with a much higher incidence in those enduring colorectal surgeries (≤45%) [1,3]. Generally, It hinders and complicates post-surgical care, resulting in higher morbidity and mortality rates [4]. According to the Centers for Disease Control (CDC), SSI is defined as a wound infection that appears either within 30 days from an operative procedure or within a year if an implant was left inside the patient, and the infection is most likely secondary to the surgical procedure [5]. The risk for surgical site infection is multifactorial, including procedure-related, patient-related, and microorganisms-related factors. SSI could affect any tissue level, such as superficial/subcutaneous, deep soft tissue, or organ spaces.

Preventative SSI measures are a vital part of surgical care to further decrease morbidity and mortality associated with it. As a result, multiple approaches such as laparoscopic surgeries have significantly reduced SSI compared to open surgeries [6]. Furthermore, oral and IV prophylactic antibiotics were proven to decrease the incidence of SSI significantly [7]. For healthcare improvement, preventive measures are being implemented to address Surgical Site Complications (SSC), which differ, ranging from hematoma, wound dehiscence, seroma, and wound infections, as previously mentioned [8]. Recently, there has been a rise in the usage of subcutaneous drains to reduce SSI incidence. Initially, subcutaneous drains were used in complex open wounds, but recently it has been finding its way towards closed wounds and incisions [1].

There is limited research investigating the impact of subcutaneous drains in colorectal surgery patients, especially in Saudi Arabia. Due to the distinct nature of colorectal surgery, it is essential to investigate the effect of subcutaneous drains on surgical site infection in colorectal surgery patients. In the current study, we primarily aim to assess the effectiveness of the placement of the subcutaneous drainage in reducing superficial SSI incidence in colorectal laparotomy surgeries. We also aim to identify the incidence of other wound complications while using subcutaneous drains.

## Materials and methods

Following this study’s scientific and ethical approval by the local institutional review board, a retrospective chart review was conducted in King Abdulaziz Medical City and National Guard Health Affairs (KAMC-NGHA), Jeddah, Saudi Arabia. The authors reviewed electronic and physical medical records of patients who underwent colorectal surgeries between the 1st of January 2015 and the 31st of December 2020. This study’s sampling technique was a non-probability consecutive sampling technique that included all eligible patients during the study period. The included colorectal surgeries were right and left hemicolectomy, sigmoidectomy, and lower anterior resection of the colon. Patients undergoing colorectal surgery at the same center KAMC-NGHA, who continued to follow up for at least 30 days postoperatively and were 16 years of age or older were included. Excluded patients were those who underwent colorectal surgery using laparoscopic techniques, open wounds, or wounds temporarily closed with vacuum-assisted closure (VAC) dressing. Subjects who met the inclusion criteria were divided into two groups: the first group included patients with subcutaneous drain, while the other group included patients without a subcutaneous drain.

The incidence of superficial SSI was measured as the primary objective, and the incidence of other complications like seromas, hematomas, and wound dehiscence were measured as the secondary objectives or outcomes. Wound assessment was done according to the CDC criteria for SSI and was filled daily by the nursing team after evaluation by the surgical team. It was flagged if diagnostic for superficial SSI in the patient’s electronic chart until SSI resolves and the wound is healed. Variables included in the analysis were patients’ demographics, pre-operative risk factors such as chronic diseases, previous abdominal surgeries, smoking status, ASA score, and colorectal cancer staging. Operative variables included were operation performed, surgery duration, prophylactic antibiotic regimen, wound contamination class, surgery urgency, and stoma presence

Quantitative or numerical variables such as age, weight, and height were presented as mean ± standard deviation. Qualitative or categorical variables such as measured clinical outcomes, chronic diseases, and previous abdominal surgeries were presented as frequency and percentages. The chi-square test or Fisher’s exact test, as appropriate, assessed the association between two categorical variables, and a p-value less than 0.05 was accepted for significance. Lastly, univariate logistic regression analysis was performed, and the goodness of fit was assessed by using the Hosmer and Lemeshow goodness test. Data were coded and entered into Microsoft Excel and exported to IBM SPSS software Version 25 (SPSS Inc, Grouponk, NY, United States) for statistical analysis.

## Results

We reviewed a total of 274 patients who underwent colorectal surgery in our hospital during the study period. However, only 208 met our inclusion criteria. The majority of the patients were non-smokers (87.5%), admitted electively (81.25%), had no previous abdominal surgeries before hospital presentation (65.87%), males (56.73%), and more than 60 years old (40.4%). Most patients had an American Society of Anesthesiologists (ASA) grade of 2 or 3, representing 40.38% and 45.19%, respectively. Out of the 208 subjects who met the inclusion criteria, the first group included 29 patients with subcutaneous drain, while the other group included 179 patients without a subcutaneous drain. Most of the drain group were between 50 and 60 years old (41.4%), whereas the other group without subcutaneous drain were mostly over 60 years old (42.5%). However, the subcutaneous drain group (when compared to the other group) had a higher proportion of colorectal cancer patients (79.3%), ASA grade 3 patients (53.6%), diabetes patients (41.4%), and hypertension patients (37.9%). On the other hand, the group without subcutaneous drain had a higher proportion of patients who were admitted electively (82.1%) had no previous abdominal surgeries (66.5%) and were males (57.5%). Furthermore, the majority of the drain group were found to have a longer length of stay exceeding 12 days (55.2%), while the group without drain mostly had a lower length of stay equal to 12 days or less (55.9%). Comparison between patients with and without subcutaneous drain are summarized in Table 1.

**Table 1:** General demographics ASA= American Society of Anesthesiologists, *Chi-square test, **Fisher’s exact test

Table 1. General demographics					
Demographics		Subcutaneous Drain, n = 29	No Subcutaneous Drain, n = 179	Total n = 208	p-value
Age	Below 50	9 (31)	49 (27.4)	58	0.29*
	50 -60	12 (41.4)	54 (30.2)	66	
	Above 60	8 (27.6)	76 (42.5)	84	
Gender	Male	15 (51.7)	103 (57.5)	118	0.557*
	Female	14 (48.3)	76 (42.5)	90	
Previous Abdominal surgeries	Yes	11 (37.9)	60 (33.5)	71	0.642
	No	18 (62.1)	119 (66.5)	137	
Smoking	Yes	3 (10.3)	23 (12.8)	26	1**
	No	26 (89.7)	156 (87.2)	182	
Hypertension	Yes	11 (37.9)	66 (36.9)	77	0.913*
	No	18 (62.1)	113 (63.1)	131	
Diabetes Mellitus	Yes	12 (41.4)	67 (37.4)	79	0.684*
	No	17 (58.6)	112 (62.6)	129	
Obese	Yes	13 (44.8)	50 (27.9)	63	0.66*
	No	16 (55.2)	129 (72.1)	145	
Colorectal cancer	Yes	23 (79.3)	133 (74.3)	156	0.563*
	No	6 (20.7)	46 (25.7)	52	
Case urgency	Elective	22 (75.9)	147 (82.1)	169	0.423*
	Emergency	7 (24.1)	32 (17.9)	39	
ASA grade	Grade 1	1 (3.6)	15 (8.6)	16	0.844**
	Grade 2	11 (39.3)	73 (42)	84	
	Grade 3	15 (53.6)	79 (45.4)	94	
	Grade 4	1 (3.6)	6 (3.4)	7	
	Grade 5	0 (0)	1 (0.6)	1	
Wound contamination class	Clean-contaminated	21 (72.4)	135 (75.4)	156	.837**
	contaminated	3 (10.3)	17 (9.5)	20	
	Dirty	5 (17.2)	27 (15.1)	32	
Blood Loss (ml)	≤250	12 (44.4)	99 (56.6)	111	0.238*
	>250	15 (55.6)	76 (43.4)	91	
Length of Surgery (mins)	≤200	10 (41.7)	68 (41)	78	0.948*
	>200	14 (58.3)	98 (59)	112	
Length of Stay (days)	≤12	13 (44.8)	100 (55.9)	113	0.268*
	>12	16 (55.2)	79 (44.1)	95	

Surgical site complications in both groups are summarized in Table 2. The overall incidence of superficial SSI among our sample was 25% (52/208). Twenty-nine patients had a subcutaneous drain, 6 of which developed superficial SSI, while 23 did not. On the other hand, 179 patients did not have a drain, 46 of which had developed superficial SSI, while 133 did not. Patients who had a subcutaneous drain had a lower incidence of seroma/hematoma and a slightly higher incidence of dehiscence than the no drain group.

**Table 2:** Outcome *Chi-square test, **Fisher’s exact test

Table 2. Outcome				
Outcomes		Subcutaneous Drain, n= 29(%)	No Subcutaneous Drain, n = 176(%)	p-value
Superficial SSI	Yes	6 (20.7)	46 (25.7)	0.563*
	No	23 (79.3)	133 (74.3)	
Seroma/Hematoma	Yes	1 (3.4)	14 (7.8)	0.70**
	No	28 (96.6)	165 (92.2)	
Dehiscence	Yes	1 (3.4)	5 (2.8)	1**
	No	28 (96.6)	174 (97.2)	

Univariate and multivariate regression analyses were performed to assess the relationship between superficial SSI development and patients’ characteristics, as illustrated in table 3. In univariate regression analysis, factors such as age, gender, previous abdominal surgery, history of diabetes, history of hypertension, history of dyslipidemia, history of obesity, history of colorectal cancer, stoma presence, ASA grade, length of surgery, blood loss, and subcutaneous drain were not predictors of superficial SSI. The analysis of factors with seeming potential to be associated with superficial SSI showed that only wound contamination class, colorectal cancer, and case urgency were of statistical significance. Multivariate subgroup analysis of the association between these factors and superficial SSI showed that wound contamination class was independently associated with superficial SSI (P= 0.049); however, the presence of colorectal cancer (P=0.338) and case urgency (P=0.465) lost their significance on multivariate analysis. Subgroup analysis comparing superficial SSI incidence in patients with and without subcutaneous drains is shown in Table 4. As can be seen, superficial SSI incidence was lower among the groups using subcutaneous drain across all variables.

**Table 3:** Risk factors relation to SSI ASA= American Society of Anesthesiologists

Table 3. Risk factors relation to SSI						
Factors		Univariate analysis		Multivariate analysis		
		Odds Ratio (95% CI)	p-value	Odds Ratio (95% CI)		p-value
Gender	Male/Female	.949 ( .504-1.787)	0.872			
Previous Abdominal Surgery	Yes/No	.724 (.366-1.434)	0.354			
smoking	Yes/No	1.711 (.712-4.115)	0.23			
Diabetes Mellitus	Yes/No	.824 (.428-1.588)	0.564			
Hypertension	Yes/No	1.086 (.569-2.073)	0.804			
Dyslipidemia	Yes/No	1.824 (.808-4.119)	0.148			
Obese	Yes/No	.707 (.347-1.440)	0.339			
Colorectal cancer	Yes/No	.413 (.209-.815)	0.011	1.619 (.604-4.337)		0.338
Case urgency	elective/emergency	.338 (.162-.704)	0.004	.465 (.207-1.042)		0.465
Stoma presence	Yes/No	.772 (410-1.454)	0.423			
ASA Grade	<2/>3	.745 (.392-1.417)	0.37			
Wound contamination class	II/>III	.366 (.186-.722)	0.004	.471 (.222-.998)		0.049
Age (Years)	<55/>56	1.114 (.586-2.115)	0.742			
Length of surgery (mins)	<200/>201	.674 (.339-1.342)	0.262			
Blood loss (ml)	<250/>251	.854 (450-1.620)	0.629			
Subcutaneous Drain	Yes/No	.754 (.289-1.968)	0.564			

**Table 4:** Subgrouping analysis ASA= American Society of Anesthesiologists, *Chi-square test, **Fisher’s exact test

Table 4. Subgrouping analysis						
factors		Subcutaneous Drain		NO Subcutaneous Drain		p-value
		SSI	No, SSI	SSI	No, SSI	
Gender	Male	3 (20%)	12 (80%)	26 (25.2)	77 (74.8%)	1**
	Female	3 (21.4%)	11 (78.6%)	20 (26.3)	56 (73.7%)	1**
Diabetes Mellitus	Yes	1 (8.3%)	11 (91.7)	17 (25.4%)	50 (74.6%)	0.278**
	No	5 (29.4%)	12 (70.6%)	29 (25.9%)	83 (74.1%)	0.772**
Hypertension	Yes	1 (9.1%)	10 (90.9%)	19 (28.8%)	47 (71.2%)	.271**
	No	5 (27.8%)	13 (72.2%)	27 (23.9%)	86 (76.1%)	.770**
Obese	Yes	1 (7.7%)	12 (92.3%)	12 (24%)	38 (76%)	.270**
	No	5 (31.3%)	11 (68.8%)	34 (26.4%)	95 (73.6%)	.766**
Colorectal cancer	Yes	3 (13%)	20 (87%)	29 (21.8%)	104 (78.2%)	.414**
	No	3 (50%)	3 (50%)	17 (37%)	29 (63%)	.664**
Elective or Emergency	Elective	4 (18.2%)	18 (81.8%)	31 (21.1%)	116 (78.8%)	1**
	Emergency	2 (28.6%)	5 (71.4%)	15 (46.9%)	17 (53.1%)	.438*
Stoma presence	Yes	4 (18.2%)	18 (81.8%)	18 (23.7%)	58 (76.3%	.774**
	No	2 (28.6%)	5 (71.4%)	28 (27.3%)	75 (72.8%)	1**
ASA Grade	<2	1 (8.3%)	11 (91.7%)	21 (23.9%)	67 (76.1%)	.293**
	>3	5 (31.3%)	11 (68.8%)	23 (26.7%)	63 (73.3%)	.763**
Wound contamination class	II	4 (19%)	17 (81%)	27 (20%)	108 (80%)	1**
	>III	2 (25%)	6 (75%)	19 (43.2%)	25 (56.8%)	.449**
Age (Years)	<55	3 (23.1%)	10 (76.9%)	18 (26.9%)	49 (73.1%)	1**
	>56	3 (18.8%)	13 (81.3%)	28 (25%)	84 (75%)	.650**
LOS (Days)	<12	2 (15.4%)	11 (84.6%)	19 (19%)	81 (81%)	1**
	>13	4 (25%)	12 (75%)	27 (34.2%)	52 (65.8%)	.475*
Length of surgery (mins)	<200	2 (20%)	8 (80%)	14 (20.6%)	54 (79.4%)	1**
	>201	3 (21.4%)	11 (78.6%)	28 (28.6%)	70 (71.4%)	.754**
Blood loss (ml)	<250	2 (16.7%)	10 (83.3%)	24 (24.2%)	75 (75.8%)	0.729**
	>251	3 (20%)	12 (80%)	21 (27.6%)	55 (72.4%)	0.751**

## Discussion

Surgical site infection complicates colorectal surgery significantly, increases morbidity, and prolongs hospital length of stay [1,2,4]. Among the areas of conflict, the use of subcutaneous drain to reduce wound complications is not well established, especially in certain patients with a higher risk of wound infection [9]. The aim of this study was to assess the incidence of surgical site complications primarily, superficial SSI, and secondarily other complications like hematoma/seroma and wound dehiscence. This study’s key findings showed that patients with a subcutaneous drain had a lower incidence of superficial SSI, seroma, and hematoma than those who did not have a drain; however, this decreased incidence was not statistically significant.

Our study’s overall incidence of superficial SSI in patients with a subcutaneous drain is 20.7% and 25.7% without drains, ranking much higher than other similar studies. In contrast, Numata et al. reported an incidence of 9.8% in patients without drainage and 3.2% in patients with a subcutaneous drain [10]. Furthermore, the incidence of seroma/hematoma and dehiscence in patients with a subcutaneous drain was the same at 3.4%. In contrast, the seroma/hematoma incidence (7.8%) was higher, and dehiscence (2.8%) was lower in patients without drains. However, no statistical significance was found between drain presence and complications. This comes in agreement with a meta-analysis done by Kosins et al., which stated that subcutaneous drains were not beneficial in prophylaxis from seroma, hematoma, or superficial SSI in abdominal wounds [11].

In the current study, superficial SSI incidence was lower among the groups using subcutaneous drain across all variables; however, the difference was statistically not significant. The overall incidence of superficial SSI among our sample was 25% (52/208). Additionally, in the subcutaneous drains group, patients who were either obese, had diabetes, hypertension, or colorectal cancer were found to have lower superficial SSI incidence (7.7%, 8.3%, 9.1%, 13%, respectively) compared to those with the same risk factors but without subcutaneous drains (24%, 25.4%, 28.8%, 21.8%, respectively); however, this decreased incidence was not statistically significant. The previous finding is not consistent with Fujii et al., where they reported a statistically significant reduction in superficial SSI rate in patients with a subcutaneous drain compared to those without (p=0.032), declining from 38.6% to 14.3%, respectively [9]. Furthermore, in agreement with the results of Fujii et al., additional factors such as age, gender, smoking, history of abdominal surgeries, and ASA grade showed no statistically significant differences in SSI reduction [9]. Nevertheless, a randomized control trial by Baier et al. demonstrated similar findings to our study showing no significant difference or benefit for subcutaneous drains over no drains [12]. The difference in outcomes is most likely related to the population's characteristics and the type of procedures in each study. Fujii et al. and Baier et al. both included variable abdominal laparoscopic surgeries that were excluded in our study. In contrast, Fujii et al. only included high-risk patients undergoing colorectal surgery [9,12].

In our population, the incidence of superficial SSI patients with class Ⅱ wounds among patients with a subcutaneous drain was 19% and 20% in patients without a drain. In comparison, superficial SSI incidence in patients with class Ⅲ or Ⅳ wounds with subcutaneous drain was 25% and 43.2% without a drain, demonstrating a substantial decrease in the incidence rate. Furthermore, our univariate regression analysis shows that wound contamination classes were independently associated with superficial SSI, specifically class Ⅲ and Ⅳ wounds, which were significantly associated with a higher risk for developing superficial SSI than class Ⅱ wounds (p=0.004). These findings are supported by Watanabe et al., who reported a rate of 15.4% in patients with class Ⅱ incision and 54.6% in patients with class Ⅲ and Ⅳ incisions, which clearly shows a substantially lower rate of superficial SSI in class ll wounds than in class Ⅲ and Ⅳ(p<0.001) [13].

Moreover, the incidence of superficial SSI in emergency surgery with a subcutaneous drain was 28.6% compared to 46.9% without drain, while patients who were admitted electively had 18.2% with a subcutaneous drain and 21.1% with no drain. Evidently, patients undergoing emergency surgery had a higher rate of superficial SSI, which was supported by univariate regression analysis showing that case urgency was independently associated with a higher risk for developing superficial SSI (p=0.004). Similar findings were reported by the studies of Zhang et al. and Alkaaki et al., which reported that emergency surgeries had a fivefold increase in the risk of development of SSI compared to elective surgeries. This could be explained by the lack of bowel prep in emergency colorectal surgeries, thus, increasing the likelihood of bacterial translocation [5,14].

There are potential limitations to this study. The first limitation is the relatively small sample size limiting the generalization of this study results. Secondly, other known risk factors that could potentially lead to superficial SSI development were not investigated. Finally, this is a single-center study that is not fully representative of the entire region. Future research should be focused on evaluating other potential predictors or risk factors for developing superficial SSI that were not mentioned in our study. A multicentric randomized controlled study with a bigger sample is needed to confirm the impact of subcutaneous drains in open colorectal surgeries.

## Conclusions

In conclusion, this study demonstrated a lower incidence of superficial SSI and seroma/hematoma in patients with a subcutaneous drain than those who did not have a drain; even though dehiscence incidence was higher in the drain group, this change in incidence was not statistically significant. Subcutaneous drains in open colorectal surgery may have a role in reducing superficial wound infection, especially in patients at higher risk for SSI. However, more prospective research is needed to prove it.
